# Prognosis in patients with coronary heart disease and breath-holding limitations: a free-breathing cardiac magnetic resonance protocol at 3.0 T

**DOI:** 10.1186/s12872-021-02402-x

**Published:** 2021-12-07

**Authors:** Keyan Wang, Wenbo Zhang, Shuman Li, Xiaoming Bi, Michaela Schmidt, Jing An, Jie Zheng, Jingliang Cheng

**Affiliations:** 1 MRI Department, The First Affiliated Hospital of Zhengzhou University, Zhengzhou, China; 2 Siemens Medical Solulations USA, Inc., Los Angeles, USA; 3 Siemens Healthcare GmbH, Erlangen, Germany; 4 Siemens Shenzhen Magnetic Resonance Ltd, Shenzhen, China; 5 Mallinckrodt Institute of Radiology, Washington University in St. Louis, St. Louis, Missouri USA

**Keywords:** Coronary heart disease, Prognoses, Free-breathing, Cardiac magnetic resonance

## Abstract

**Background and purpose:**

Conventional cardiac magnetic resonance (CCMR) imaging is usually performed with breath-holding (BH), which is adverse in patients with BH limitations. We explored the ability of a free-breathing CMR (fCMR) protocol to prognosticate in patients with coronary heart diseases (CHD) and limited BH ability.

**Methods:**

Sixty-seven patients with CHD and limited BH abilities were prospectively enrolled in this study. All patients underwent comprehensive fCMR imaging at 3.0 T. The fCMR protocols included compressed sensing (CS) single-shot cine acceleration imaging, and motion-corrected (MOCO), single-shot late gadolinium enhancement (LGE) imaging. Image quality (IQ) of the cine and LGE images was evaluated based on the 5-point Likert scale. The value of fMRI in providing a prognosis in patients with CHD was assessed. Statistical methods included the T test, Mann–Whitney test, Kappa test, Kaplan–Meier curve, Log-rank test, Cox proportional hazard regression analysis, and receiver operating characteristic curves.

**Results:**

All IQ scores of the short axis CS-cine and both the short and long axes MOCO LGE images were ≥ 3 points. Over a median follow-up of 31 months (range 3.8–38.2), 25 major adverse cardiovascular events (MACE) occurred. In the univariate analysis, infarction size (IS), left ventricular ejection fraction (LVEF), 3D-Global peak longitudinal strain (3D-GPLS), heart failure classification were significantly associated with MACE. When the significantly univariate MACE predictors, added to the multivariate analysis, which showed IS (HR 1.02; 95% CI 1.00–1.05; *p* = 0.048) and heart failure with preserved EF (HR 0.20; 95% CI 0.04–0.98; *p* = 0.048) correlated positively with MACE. The optimal cutoff value for LVEF, 3D-GPLS, and IS in predicting MACE was 34.2%, − 5.7%, and 26.1% respectively, with a sensitivity of 90.5%, 64%, and 96.0% and specificity of 72%, 95.2%, and 85.7% respectively.

**Conclusions:**

The fCMR protocol can be used to make prognostic assessments in patients with CHD and BH limitations by calculating IS and LVEF.

## Background

Coronary heart disease (CHD) is primarily caused by coronary atherosclerosis, which causes coronary artery stenosis and myocardial ischemia. These changes then lead to myocardial infarction (MI) or sudden death. Cardiac magnetic resonance (CMR) imaging has been used to diagnose MI for a long time [1–3]. Previous studies have demonstrated that the CMR markers of myocardial and microvascular damage add incremental prognostic information to clinical diagnoses [4–7]; however, most of these studies lack prognostic information in patients with CHD and BH impairments. An absence of accepted CMR marker cutoff values to enable optimized risk stratifications in clinical practice is also lacking. Conventional CMR (CCMR) protocols used in our center primarily include routine cine and late gadolinium enhancement (LGE) imaging. Data acquisitions are typically performed with breath-holding (BH). In clinical practice, a few of patients with CHD quit CCMR examinations because it was difficult to perform BH in the time needed.

Accelerated single-shot methods for cine imaging using compressed sensing (CS) acceleration [8] and LGE imaging using motion correction (MOCO) techniques are accepted for free-breathing imaging [9]. CS cine imaging has shown good consistency for assessing left ventricular function with standard cine protocols, and MOCO LGE has shown good consistency for MI detection with conventional LGE [10–13]. Although these two techniques had high consistency in cardiac functional evaluations and myocardial infarction detection compared with traditional techniques, no research has been performed on the application of these two techniques to prognosticate patients with CHD and BH limitations. This study aimed to develop a comprehensive free-breathing cardiac magnetic resonance (fCMR) protocol, including CS cine and MOCO LGE sequences, for patients with CHD and BH limitations. We also sought to explore the value of this protocol in determining prognoses in patients with CHD and BH limitations.

## Methods

### Subject enrollment

The institutional review board of our institution approved this study. We prospectively recruited adult patients scheduled for CMR examinations from February 1, 2017, to June 18, 2019. The inclusion criteria were: (1) Patients with CHD confirmed with DSA, who also had BH limitations and could not undergo CCMR protocols, including those with severe heart failure and coma, and other vulnerable patients; (2) A glomerular filtration rate of ≥ 30 mL/min per 1.7m^2^, and no other contraindications for CMR imaging. DSA images were analyzed for stenosis by a radiologist with greater than 10 years of experience. According to the criteria prognostically significant for CHD, CHD was diagnosed if vessel stenosis was ≥ 70% on DSA [14]. We obtained patient baseline characteristics from the electronic medical records.

### The CMR imaging protocol

**Fig. 1:**
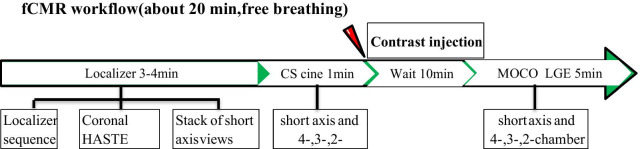
The workflow of the free-breathing cardiac resonance (fCMR) protocol. *HASTE* half-Fourier singlc-shot turbo spin-echo, *MOCO LGE* motion-corrected, single-shot late gadolinium enhancement, *CS* compressed sensing

fCMR scans were performed on a clinical 3 T MRI scanner (MAGNETOM Skyra, Siemens Healthcare, Erlangen, Germany). The system was equipped with an 18-element body array coil and a 32-element spine array coil. The fCMR protocol primarily included: (1) CS cine imaging with balanced steady-state free precession (bSSFP) readout, featuring a two-dimensional, real-time, true fast imaging, steady-state precession, sparse data sampling and iterative reconstruction (SSIR). (2) MOCO LGE is characterized by respiratory motion-corrected, single-shot steady-state free procession, and averaged phase-sensitive inversion recovery (PSIR). These parameters are shown in Table 1. CS cine images were performed in three long-axis (LAX) planes (four-, three-, and two-chamber planes) and a stack of short-axis (SAX) images covering the left ventricle (LV). MOCO LGE sequence images were acquired in the same SAX and LAX planes as the CS cine images. Intravenous gadolinium diethylenetriamine penta-acetic acid (Gd-DTPA) contrast agent was administered at a dose of 0.2 ml/kg of body weight. MOCO LGE images were acquired 10 min after contrast injection. The workflow is shown in Fig. 1.

**Table 1:** Typical free-breathing cardiac resonance (fCMR) image acquisition parameters

Parameters	MOCO-LGE LAX	MOCO-LGE SAX	CS-cine SAX	CS-cine LAX
Echo time (ms)	1.18	1.18	1.2	1.2
Repetition time (ms)	2.8	2.9	2.8	2.8
Temporal resolution (ms)	420	–	42.3	42.6
Spatial resolution reconstructed (mm^3^)	1.4 × 1.4 × 6.0	1.4 × 1.4 × 8.0	1.7 × 1.7 × 8.0	1.7 × 1.7 × 6.0
Bandwidth (Hz/pixel)	1085	1085	910	962
Section thickness (mm)	6	8	8	6
Section gap (mm)	1.2	1.6	1.6	1.2
No. of sections	3	7–10	7–10	3
Flip angle (degrees)	40	50	50	50
Breath holds (n)	0	0	0	0
Acquisition time (s)	37 ± 6	120 ± 19	25 ± 5	8 ± 2
Accelerate factor	8	8	20	20
ECG mode	Prospective triggering	Prospective triggering	Adaptive triggering	Adaptive triggering

## Image analysis

### Image quality

All fCMR images were transferred to a workstation (cmr42, Circle Cardiovascular imaging, Version 5.12.1, Calgary, Alberta, Canada) for evaluation. The IQ of CS cine and MOCO LGE images were evaluated by a single reviewer with > 5 years of experience in cardiovascular imaging diagnostic. For a randomly selected sample of 30 patients, the reproducibility of the IQ scores was evaluated by another senior observer. IQ scores were evaluated based on the 5-point Likert score ranging from 1 to 5 (5 = excellent image quality, 4 = normal image quality, 3 = presence of artifacts but sufficient image quality, 2 = severe artifacts in the area of the left ventricle, and 1 = completely nondiagnostic images) [11, 12, 15].

### CS cine imaging

Left ventricular function (LVF) and feature tracking (FT) were measured with the cmr42 software (version 5.12.1, Circle Cardiovascular Imaging, Calgary, Canada), including the LV ejection fractions (LVEF), LV end-diastolic mass (LVEDM), and 3D-Global peak longitudinal strain (3D-GPLS). The end-systolic and end-diastolic phases were detected automatically at the endocardium and epicardium contours, which were automatically delineated on the short-axis CS cine images with software and were based on the smallest and largest LV volumes over the entire cardiac cycle. Contours rendered by automated analyses were reviewed and manually adjusted whenever needed [10]. LV papillary muscles and trabeculations were included in ventricular cavity volumes [16, 17]. For FT quantifications, LV epicardial and endocardial borders were automatically or manually adjusted where needed and delineated at end-diastole of the short axis and at the 2ch, 3ch, and 4ch long-axis cine images, and propagated. The 3D-GPLS values were derived in accordance with the American Heart Association’s 16-segment model, excluding the apical cap, because the apex is not beneficial in observing short-axis slices, and the measurement error is relatively large.

### MOCO LGE

The presence of LGE involving the subendocardium of stenotic coronary arterial distributions was classified as ischemic LGE, otherwise, it was classified as non-ischemic LGE [3, 18, 19]. The presence and patterns of LGE were visually assessed on the short- and long-axis images using the American Heart Association (AHA) 17-segment model. Segments 1, 2, 3, 7, 8, 13, 14, and 17 are supplied with blood through the left anterior descending (LAD) artery; segments 5, 6, 11, 12, and 16 are supplied through the left circumflex artery (LCX); and segments 4, 9, 10, and 15 are supplied through the right coronary artery (RCA). Short-axis LGE image stacks with minimal through-plane motion were used to quantify the pixel-wise infarct size (IS) [13]. Computer-assisted planimetry using the cmr42 software quantified the IS, using the full width at half-maximum technique, an accepted technique for LGE quantification [20].

## Follow-up

Clinical outcomes were collected during follow-up. The primary clinical outcome of the study was the composite endpoint of major adverse cardiac events (MACE), defined as a composite of all-cause mortality, and new heart failure diagnosis. All-cause death was defined as any death during the follow-up period. Heart failure (HF) was defined as clinical syndromes with symptoms and/or signs caused by a structural and/or functional cardiac abnormality and corroborated by elevated natriuretic peptide levels and/or objective evidence of pulmonary or systemic congestion [21]. Outcome data were collected from scheduled study follow-up visits, monthly telephone calls, and reviews of the electronic case records.

## Statistical analyses

Statistical analyses were performed using the dedicated software, SPSS 20.0 (SPSS Inc., Chicago, USA) and and MedCalc10.0 (MedCalc Software, Ostend, Belgium) software. Continuous data were checked for normality using the Shapiro–Wilk test and presented as the mean ± standard or median (interquartile range [IQR]). Qualitative variables were expressed as percentages. The Kappa statistic was used to evaluate the consistency between readers 1 and 2 for the IQ assessments. Kaplan–Meier survival analysis was used to analyze qualitative variables associated with MACE, reporting *X*^2^. Cox proportional hazards regression analysis was used to analyze continuous variables associated with MACE, reporting hazard ratios (HRs) and 95% confdence intervals (CIs). In our study, because the sample was small, variables showing values of *p* < 0.01 on univariate analysis were entered into multivariate Cox proportional hazard regression analysis. Cumulative survival rate and rate of MACE were estimated using a Kaplan–Meier curve evaluated by the log-rank test. Receiver operating characteristics (ROC) curves, used to derive the optimal cutoff points of LVEF, 3D-GPLS, and IS, forecasted the MACE from the non-MACE group. Areas under the curve (AUC), sensitivities, and specificities were respectively calculated. Probability values were 2-sided, and values of *p* < 0.05 were considered significant.

## Results

### Patient characteristics

Over two years, from February 1, 2017, to June 18, 2019, a total of 9256 patients underwent CMR imaging in our department; 67 (0.72%) patients were diagnosed with CHD by DSA and unable to meet the demands of multiple BH sessions and long examination times. Of these 67 enrolled patients, 35 had single coronary artery stenosis, 17 had two coronary artery stenoses, 15 cases had three coronary artery stenoses. 51 cases had LAD stenoses, and the mean percentage of patients with stenosis was 90% (85–100%). 33 patients had LCX stenoses, and the mean percentage of patients with stenosis was 80% (75–90%). 30 patients had RCA stenoses, and the mean percentage of patients with stenosis was 95% (80–100%). The demographic characteristics of the cohort are shown in Table 2.

**Table 2:** Demograohic characteristics of the study cohort

Demographic characteristics	CHD (n = 67)
Age, years	59 ± 12
Male, n (%)	49 (73.1)
Body mass index, kg/m^2^	1.74 ± 0.22
Resting heart rate > 100 bpm, n (%)	0 (0)
History of hypertension, n (%)	35 (52.2)
History of diabetes, n (%)	21 (31.3)
History of hypercholesterolemia, n (%)	18 (26.9)
Heavy tobacco use, n (%)	25 (37.3)
Family history of CHD, n (%)	14 (20.9)
Percutaneous coronary intervention, n (%)	42 (62.7)
Cardiac bypass surgery, n (%)	2 (3.2)
β-Blocker, n (%)	53 (79.1)
Calcium channel blocker, n (%)	49 (73.1)
ACE inhibitor, n (%)	25 (32.5)
Aspirin, n (%)	67 (100)
Statin, n (%)	60 (89.6)
Nonsinus rhythm, n (%)	0 (0)
Left bundle-branch block, n (%)	9 (13.4)
Right bundle-branch block, n (%)	3 (4.5)
Troponin, ng/ml	0.09 (0.01 to 1.06)
BNP, pg/ml	726 (336 to 1550)
Left ventricular ejection fraction, %	43.5 ± 19.0
Left ventricular end-diastolic volume, ml	167.7 (124 to 223.9)
Left ventricular end-systolic volume, ml	87.5(53.3 to 152.9)
Left ventricular stroke volume, ml	69.9 ± 27.8
Left ventricular end-diastolic mass, g	117.7 ± 35.3
3D-Global peak longitudinal strain, %	− 8.5 (− 5.4 to − 11.2)
Infarction size, %	22.4 (9 to 40)
Heart failure with preserved EF, n (%)	20 (29.9)
Heart failure with mid-range EF, n (%)	22 (32.8)
Heart failure with reduced EF, n (%)	25 (37.3)
Left anterior descending stenosis, %	90 (85–100)
Left circumflex artery stenosis, %	80 (75–90)
Right coronary artery stenosis, %	95 (80–100)

### Image quality

**Fig. 2:**
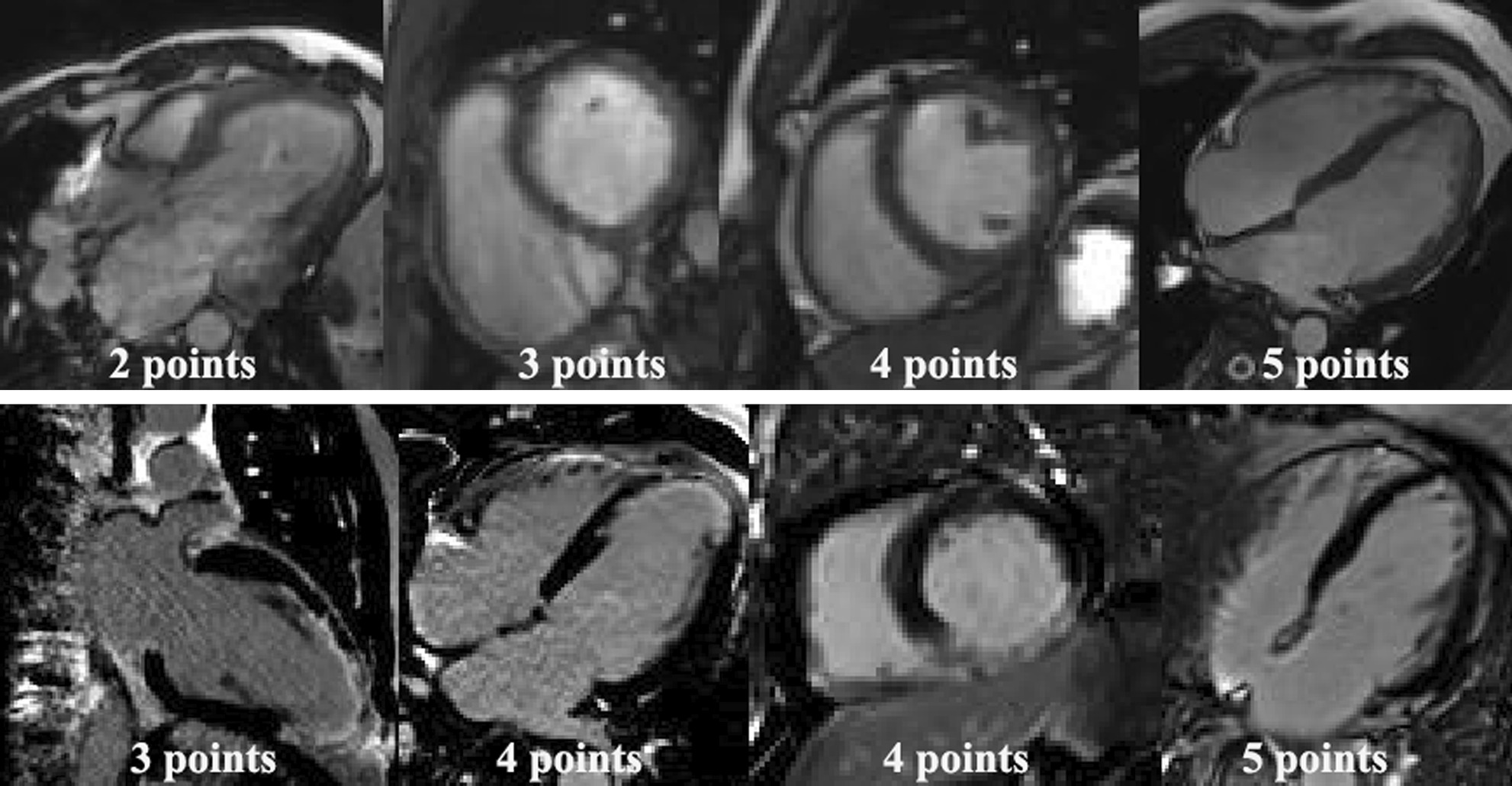
Typical images display with different scores ranging from 2 to 5. 2 points: sever artifact; 3 points: presence of artifacts but acceptable; 4 points: good; 5 points: excellent. Our results lack images with an image quality score of 1 point

The SAX CS cine image quality scores were 3 (50.5%), 4 (39.6%), and 5 (9.9%) points, and the median IQR was 3 (3–4) points. The LAX CS cine image quality scores were 2 (18.2%), 3 (46.8%), 4 (33.2%), and 5 (1.8%) points, and the median IQR was 3 (3–4) points. The SAX MOCO LGE image quality scores were 3 (10.8%), 4 (15.3%), and 5 (73.9%) points, and the median IQR was 5 (4–5) points.The LAX MOCO LGE image quality scores were 3 (12.6%), 4 (55.9%), and 5 (31.5%) points, and the median IQR was 4 (4–5) points. Readers 1 and 2 had good concordance in their decisions on the IQ assessments; SAX CS cine [IQ, kappa:0.80, 95% CI (0.70–0.92)], LAX CS cine [IQ, kappa:0.72, 95%CI (0.67–0.90)], SAX MOCO LGE [IQ, kappa:0.94, 95%CI (0.89–1.00)], and LAX MOCO LGE [IQ, kappa:0.85, 95%CI (0.78–0.92)]. Typical cases of IQ points is shown in Fig. 2.

## fCMR parameters

**Fig. 3:**
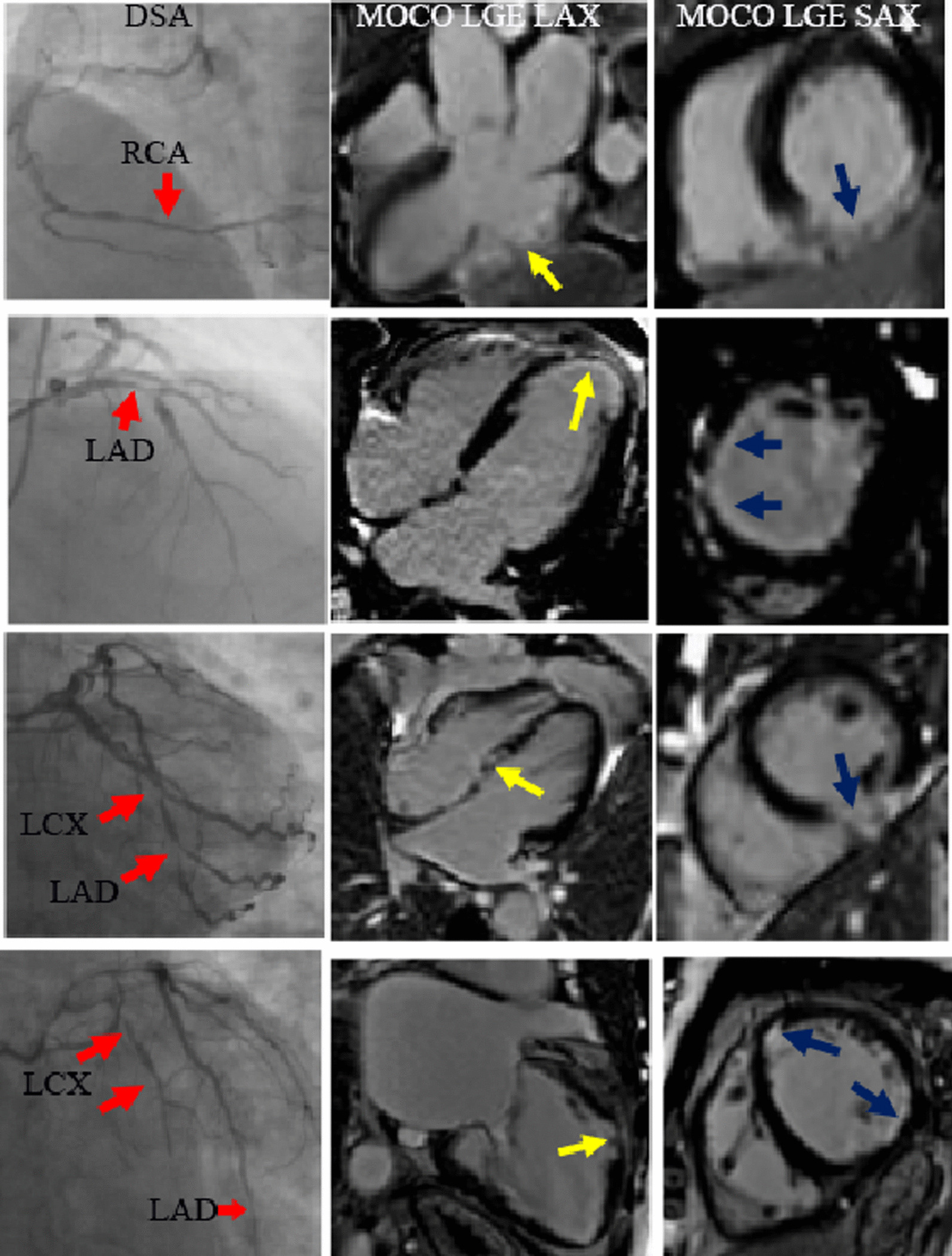
Representative images of patients with coronary heart disease, including SAX and LAX MOCO LGE, and DSA. *LAD* left anterior descending artery, *LCX* left circumflex artery, *RCA* right coronary artery, *SAX* short-axis, *LAX* long-axis, *MOCO LGE* motion-corrected late gadolinium enhancement, *DSA* digital subtraction angiography

After reviewing fCMR findings in the study, MOCO LGE detected 62 cases with positive ischemic LGE, 15 cases had non-ischemic LGE. Representative cases are shown in Fig. 3. The mean fCMR parameters value of 67 subjects were as follow: infarction size 22.9% (9 to 40%), LVEF 43.5% ± 19.0%, LVEDV 167.7% (124 to 223.9%), LVESV 87.5% (53.3 to 152.9%), LVSV 69.9% ± 27.8%, LVEDM 117.7 g ± 35.3 g, 3D-GPLS − 8.5% (− 5.4 to − 11.2%). 67 patients were divided into 3 groups according 2021 ESC Guidelines for the diagnosis of heart failure [21]: 20 patients were diagnosed as heart failure with preserved EF (HFpEF; defined as EF ≥ 50%), 22 patients were diagnosed as heart failure with mid-range EF (HFmrEF; an EF of 40–49%), 25 patients were diagnosed as heart failure with reduced EF (HFrEF; defined as EF < 40%).

## Survival analyses@@@

All the enrolled patients completed follow-up over a median of 31 months (3.8 to 38.2 months), 25 composite events (37.3%, 10 deaths, and 15 hospitalizations due to recurrence of heart failure) were observed.

Cox proportional hazards regression analysis was performed to investigate associations between baseline characteristics, fCMR parameters and MACE (Table 3). In the univariate analysis, IS (hazard ratio [HR] 1.05; 95% confidence interval [CI] 1.03–1.07; *p* < 0.001), LVEF (HR 0.94; 95% CI 0.92–0.94; *p* < 0.001), 3D-GPLS (HR 1.15; 95% CI 1.09–1.29; *p* < 0.001), LVEDM, heart failure, age, diabetes mellitus, hypertension, ACE inhibitor use were significantly associated with MACE. LVEDV, LVESV, and LVSV were used to calculate LVEF. Therefore, LVEDV, LVESV, and LVSV were not incorporated into the univariate and multivariate analysis. According to the rule of thumb for multivariate analyses, 25 incidents were recorded during the follow-up, maximum 4 variables should be included in the multivariate analysis. The purpose of the study was to evaluate the value of fCMR parameters in the prognosis of patients with CHD and BH limitation, three fCMR parameters including IS, LVEF and 3D-GPLS, and HF classification were chose in the multivariate analysis. Multivariate analysis showed IS (HR 1.02; 95% CI 1.00–1.05; *p* = 0.048) and HFpEF (HR 0.20; 95% CI 0.04–0.98; *p* = 0.048) correlated positively with MACE.

**Table 3:** Cox proportional hazards regression analysis for MACE

Univariate predictors	HR/*X*^2^	95.0% CI	*P*-value
IS (per 1% increase)	1.05	1.03–1.07	< 0.001
LVEF (per 1% increase)	0.94	0.92–0.96	< 0.001
LVEDM (per 1 g increase)	1.01	1.00–1.02	0.032
3D-GPLS (per 1% decrease)	1.15	1.09–1.29	< 0.001
Heart failure classification	36.2		< 0.001
Male gender	0.01		0.929
Age (per 1 year increase)	1.06	1.03–1.09	0.030
Diabetes mellitus	4.24		0.039
Hypertension	8.13		0.004
Hyperhomocysteinemia	0.08		0.783
Smoker	0.00		0.989
PCI	3.26		0.071
Aspirin use	3.14		0.076
ACE inhibitor use	4.43		0.035
Beta-blocker use	3.74		0.053
Calcium channel blocker use	1.41		0.235
Statin use	0.50		0.478

Multivariate predictors	HR	95.0% CI	*P *value
IS (per 1% increase)	1.02	1.00–1.05	0.048
LVEF (per 1% increase)	1.03	0.96–1.09	0.428
3D-GPLS (per 1% decrease)	1.15	0.92–1.44	0.199
HFpEF	0.20	0.04–0.98	0.048

*IS* infarction size, *LVEF* left ventricular ejection fraction, *LVEDV* left ventricular end-diastolic volume, *LVESV* left ventricular end-systolic volume, *LVSV* left ventricular systolic volume, *LVEDM* left ventricular end-systolic mass, *3D-GPLS* three-dimensional global peak longituditial strain, *PCI* percutaneous coronary intervention, *HF* hear failure, *HFpEF* heart failure with preserved EF

All three fCMR parameters had reasonable accuracy in predicting MACE. The optimal cutoff value for LVEF in predicting MACE was 34.2%, with a sensitivity of 90.5% and specificity of 72%. The optimal cutoff value for 3D-GPLS in predicting MACE was − 5.7%, with a sensitivity of 64% and specificity of 95.2%. The optimal cutoff value for IS in predicting MACE was 26.1%, with a sensitivity of 96.0% and specificity of 85.7%. The AUC for LVEF, 3D-GPLS, IS was 0.854, 0.786, and 0.703, respectively (all, *p* < 0.05).

**Fig. 4:**
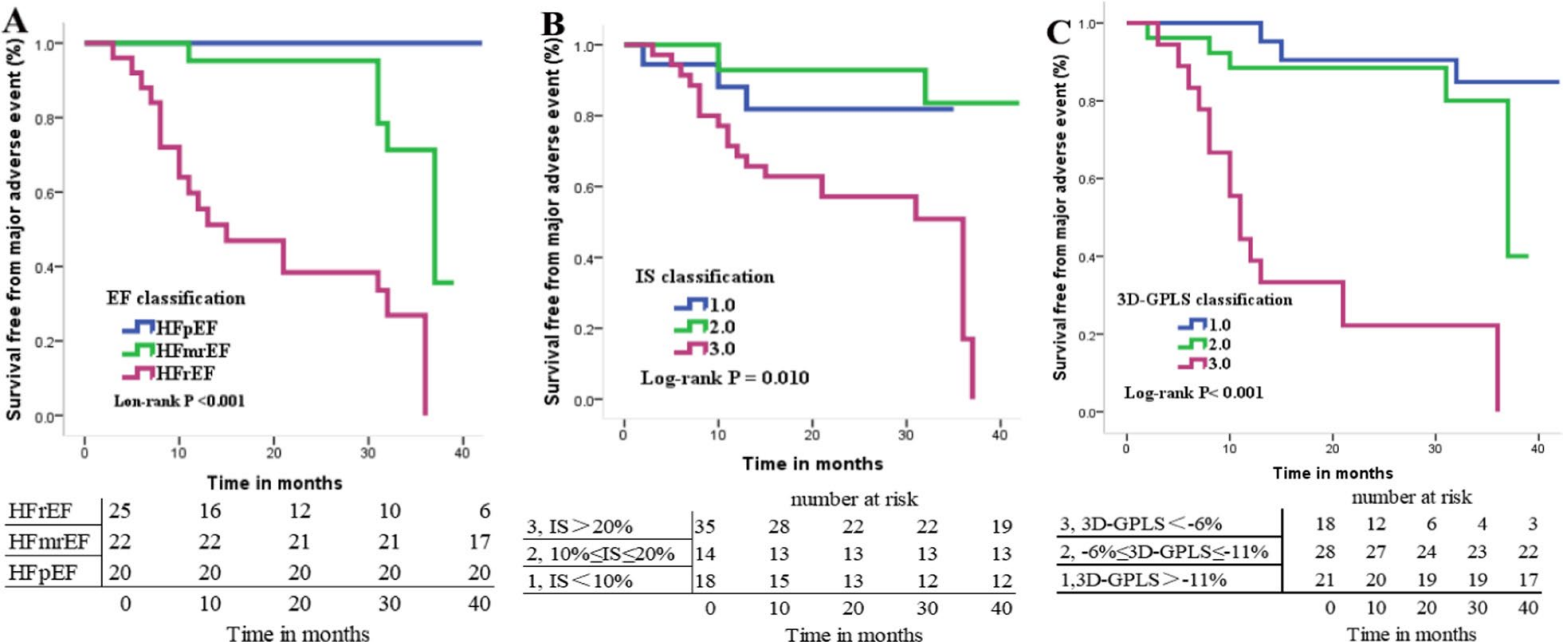
Kaplan–Meier analysis of survival rates based on free-breathing cardiovascular magnetic imaging parameters. **A** The hazard ratio of patients without heart failure (HF) but normal EF groups, HFpEF, HFmrEF and HFrEF. **B** The hazard ratio of IS with different grades. **C** The hazard ratio of 3D-GPLS with different grades. 3D-GPLS, three-dimensional global peak longituditial strain; *HFrEF* heart failure with preserved ejection fraction (EF), *HFmrEF* heart failure with mid-range EF, *HFrEF* heart failure with reduced EF, *IS* infarction size

Kaplan–Meier analyses showed a significant and progressive decrease in survival rates in the patients with HFpEF group, followed by the HFmrEF group, and then by the HFrEF group (*p* < 0.001) (Fig. 4A). The Kaplan–Meier analysis showed a significantly lower survival rate in the IS $>$ 20% group than in the IS $\le$ 20% group (*p* = 0.010)(Fig. 4B), and a significantly lower survival rate in the 3D-GPLS $<$ − 6% group than in the 3D-GPLS $\ge$ − 6% group (*p* < 0.001) (Fig. 4C).

## Discussion

The data in this study was derived from patients with CHD and BH limitation who could not perform CCMR examinations and used fCMR as an alternative examination. The fCMR protocol obtained robust images and could be used to perform prognostic assessments of patients with CHD and BH limitation. This protocol can be used as an alternative imaging technique for patients with breath-holding restrictions.

In the study, CCMR imaging was not performed on patients who could not hold their breath for an extended amount of time since the CCMR acquisitions required multiple breath-holds to avoid respiratory image artifacts [22, 23]. Also, to achieve sufficiently high spatial and/or temporal resolutions during CCMR imaging, segmented k-space data are acquired over multiple heartbeats resulting in segmented data acquisitions that are prone to motion artifacts, leading to suboptimal image quality. Motion artifacts on CCMR images of patients with BH limitations are widely recognized, meaning that CCMR imaging in these patients has mostly been abandoned. Single-shot readout protocols effectively eliminate breathing motion artifacts in both CS cine [24–26] and MOCO LGE images [27]. Moreover, MOCO LGE is characterized as a protocol that provides motion correction and has allowed the fCMR protocol to obtain robust images under free-breathing, which has been confirmed by our study.

Generally, the validation of novel techniques is often performed through comparative studies with reference techniques considered as the gold standard. For LVF quantification, standard segmented BH cine imaging is the most accurate and reproducible imaging technique [10, 12]. However, this method cannot be performed in our patients with BH limitations. Therefore, in this study, we showed that almost all CS cine imaging yielded robust SAX images; the high IQ scores of the CS cine images translated into high reliability for measuring left ventricular function and strain. A few LAX CS cine images had poor IQ scores, similar to what has been previously published [28], which could have been due to flow-related artifacts occurring in the phase-encoding direction during systole since the temporal domain sparsity might be limited in the anatomic regions where there is very high flow [29]. LGE have been widely used to detect subendocardial infarcts in patients with CHD, determine the extent of MI, and provide diagnostic information for the treatment and prognosis of patients with CHD [4, 30]. Previous studies have reported no differences in MI detection between MOCO LGE and conventional LGE [13, 28]. Although we did not obtain conventional LGE imaging, all MOCO LGE imaging yielded diagnostic images. In our study, fifteen patients with CHD confirmed by DSA were diagnosed as non-ischemic LGE on MOCO LGE imaging. This negative finding after MOCO LGE imaging could be explained by incomplete cardiac coverage, missed small subendocardial LGE due to poor contrast with the blood pool [31], or good compensation and no MI occurrence in the patients with CHD.

In this study, 37.3% of patients experienced MACE during a median follow-up of 31 months. This incidence of MACE is higher than in previous studies [4–7] and is likely because all patients in the study had BH impairments, and most were vulnerable. Our study was first to show that HFpEF, and IS derived from MOCO LGE, had independent values in predicting MACE in patients with CHD and BH limitation. LVEF remains the primary parameter for HF characterization and the primary inclusion criterion for clinical trials of HF [32]. The LVEF ≤ 35% derived from standard segmented BH cine imaging has been recognized as a strong predictor of adverse outcomes [5, 7]. In our study, the optimal cutoff value for LVEF, derived from CS cine, in predicting MACE was 34.2%, with a high sensitivity but low specificity. Considering the multiple mechanisms involved in MACE, it seems unlikely that LVEF will provide adequate prognosis information for all patients [5]. Comprehensive CMR stratification tools, including 3D-GPLS and IS, could improve risk stratification significantly. 3D-GPLS is, perhaps, a more sensitive measure of myocardial contractile function than LVEF [4]. In our study, the optimal cutoff value for 3D-GPLS and IS in predicting MACE was − 5.65% and 26.1%, respectively. 3D-GPLS had high specificity but low sensitivity, whereas IS had both high sensitivity and specificity. LVEF, 3D-GPLS, and IS have individual advantages in providing prognoses and can complement one another. The benefit of the fCMR protocol is not limited to a patient’s BH ability, broadening CMR imaging applications and allowing the realization of imaging goals in patients with BH limitations. The fCMR protocol has been successfully applied to the prognostic evaluations of patients with CHD, making up for the deficiency of CCMR imaging while allowing patients the opportunity to obtain CMR images. Therefore, cardiologists can understand the occurrence, development, and prognosis of patients with CHD and BH limitation, and further to give accurate and individualized treatments.

There were some limitations to this study. First, it was a single-center study and, therefore, reflects the use of clinical protocols at our institution. Another major limitation is the lack of CCMR imaging, and due to practical reasons, fCMR imaging was systematically performed when CCMR methods could not be implemented, which could have selected for a group that benefited most from fCMR imaging. The sample size was also small, and the follow-up time was short. Future studies should be performed with larger sample sizes and longer follow-up times. Moreover, patients with CHD should have received optimal medical treatments according to the hospital guidelines, such as receiving the combination medical regimens, including sacubitril/valsartan. In our study, the absence of assessing the use of these medications is a limitation for risk assessments. Finally, the fCMR protocol did not include advanced MR sequences, such as mapping, perfusion, and blood flow. We did not evaluate the incremental benefit of T2-weighted STIR imaging for myocardial edema because an optimized sequence in currently not available.

## Conclusions

For patients who were unable to undergo CCMR imaging, the fCMR protocols obtained robust images that could be used to evaluate cardiac function and detect myocardial infarction, and could be used to perform prognostic assessments. This protocol is promising as an alternative technique to CCMR and could replace CCMR in the future.

## Data Availability

The datasets generated for this study are available on reasonable request to the corresponding author.

## Abbreviations

LGE: Late gadolinium enhancement
IS: Infarction size
LV: Left ventricle
LVEDV: Left ventricular end-diastolic volume
LVEF: Left ventricular ejection fraction
LVESV: Left ventricular end-systolic volume
LVSV: LV stroke volumes
CMR: Cardiac magnetic resonance
ECG: Electrocardiogram
LVF: Left ventricular function
FT: Feature tracking
3D-GPLS: 3D-Global peak longitudinal strain
CHD: Coronary heart diseases
BH: Breath-holding
fCMR: Free-breathing CMR
MOCO: Motion-corrected
CS: Compressed sensing
IQ: Image quality
MACE: Major adverse cardiovascular events
MI: Myocardial infarction
AHA: American Heart Association
LAD: Left anterior descending
LCX: Left circumflex artery
RCA: Right coronary artery
ROC: Receiver operating characteristics
AUC: Area under the curve
LAX: Long-axis
SAX: Short axis
Gd-DTPA: Gadolinium diethylenetriamine pentaacetic acid
bSSFP: Balanced steady-state free procession
SSIR: Sparse data sampling and iterative reconstruction
